# High prevalence and poor linkage to care of transfusion‐transmitted infections among blood donors in Dar‐es‐Salaam, Tanzania

**DOI:** 10.1111/jvh.13073

**Published:** 2019-02-27

**Authors:** Zameer Mohamed, Jin U. Kim, Alex Magesa, Mabula Kasubi, Sarah F. Feldman, Stephane Chevaliez, Promise Mwakale, Simon D. Taylor‐Robinson, Mark R. Thursz, Yusuke Shimakawa, John Rwegasha, Maud Lemoine

**Affiliations:** ^1^ Department of Hepatology Imperial College London St Mary's Hospital London UK; ^2^ Department of Biochemistry and Haematology Muhimbili National Hospital Dar es Salaam Tanzania; ^3^ Unité d’Épidémiologie des Maladies Émergentes Institut Pasteur Paris France; ^4^ French National Reference Center for Viral Hepatitis B, C and delta Department of Virology Hopital Henri Mondor Université Paris‐Est Créteil France; ^5^ Department of Bioethics Muhimbili University of Health and Allied Sciences Dar es Salaam Tanzania; ^6^ Department of Gastroenterology Muhimbili National Hospital Dar es Salaam Tanzania

**Keywords:** blood donation, linkage to care, sub‐Saharan Africa, transfusion‐transmitted infections (TTIs)

## Abstract

Blood transfusion is one of the most commonly relied upon therapies in sub‐Saharan Africa. Existing safeguards recommended include systematic screening for transfusion‐transmitted infections and restricted voluntary nonremunerated blood donor selection. We report the transfusion‐transmitted infection screening and notification practice at a large urban blood transfusion centre in Dar‐es‐Salaam, Tanzania. Between October 2016 and March 2017 anonymized records of all donors registered at the blood transfusion unit were accessed to retrospectively note demographic information, donor status, first‐time status, transfusion‐transmitted infection result and notification. 6402 consecutive donors were screened for transfusion‐transmitted infections; the majority were family/replacement blood donors (88.0%) and male (83.8%). Overall transfusion‐transmitted infections prevalence was 8.4% (95% CI 7.8‐9.1), with hepatitis B being the most prevalent infection (4.1% (95% CI 3.6‐4.6)). Transfusion‐transmitted infections were more common in family/replacement blood donors (9.0% (95% CI 8.3‐9.8)) as compared to voluntary nonremunerated blood donor (4.1% (95% CI 2.8‐5.7)). A minority of infected‐donors were notified of a positive result (8.5% (95% CI 6.3‐11.2)). Although transfusion‐transmitted infections are more prevalent among family/replacement blood donors, overall risk of transfusion‐transmitted infections across all groups is considerable. In addition, existing efforts to notify donors of a positive transfusion‐transmitted infection are poor. Future policies must focus on improving linkage to care for newly diagnosed patients with transfusion‐transmitted infections.

**Abbreviations**
FRD: family/replacement blood donors
HBV: hepatitis B virus
HCV: hepatitis C virus
MNH: Muhimbili National Hospital
SSA: sub‐Saharan Africa
TTI: transfusion‐transmitted infections
VNRD: voluntary nonremunerated blood donors

## 1 INTRODUCTION

Blood transfusion forms part of the backbone of basic medical care in sub‐Saharan Africa (SSA), in particular, its use is crucial in limiting mortality associated with malaria and obstetric blood loss.^1^ Its importance is further underlined by its inclusion on the World Health Organization (WHO) model list of essential medicines.^2^

Despite the well‐documented demand for blood transfusion, there is a chronic shortage of supply in SSA. In 2013, the WHO estimated that of the 112.5 million blood donations globally, only 5.6 (5%) were donated in SSA. This translates to less than 4 units per 1000 people in SSA, compared to more than 35 units per 1000 people in Europe, languishing behind the WHO minimum target of 10 units per 1000 people.^3^

In addition to challenges to supply, minimizing the risk of transfusion‐transmitted infections (TTIs ie HIV, hepatitis B (HBV) and hepatitis C (HCV) viruses and syphilis) is imperative. Blood donor recruitment policy varies vastly across the globe, which has an impact on blood transfusion safety. Well‐resourced settings are primarily serviced by altruistic repeat donors, who are considered low risk.^3^ In comparison, it is reported that more than 50% of WHO Africa region countries are dependent on replacement donors (commonly relatives or financially incentivized donors), who are considered to be higher risk of TTIs.^1^

Despite these risks, blood products are not systematically screened prior to transfusion in SSA.^4^, ^5^ In an attempt to address these issues, the WHO regional office for Africa released its strategy for improving blood transfusion quality and adequacy for the region by 2012.^6^ This included 75% of member states having a national blood transfusion policy, 100% of donations being screened for TTIs and having at least 80% of donation from voluntary nonremunerated blood donors (VNRD).^6^ Compliance with these directives has been variable among the African member states. For example, in Tanzania like in many sub‐Saharan African countries, there remains a heavy reliance on family/replacement blood donors (FRD); however, Tanzania has implemented a national blood transfusion policy and systematic screening of TTIs.^7^

Screening for TTIs also provides an important opportunity for notification and linkage to care of donors. As part of its HIV and viral hepatitis elimination strategies, the WHO has set out ambitious objectives to improve diagnosis and care for HIV and viral hepatitis.^8^, ^9^ Thus, TTI positive blood donors are an ideal population to transition to specialist care. To date, there has been minimal description of TTI linkage to care in blood transfusion settings in SSA, with existing reports limited to West African studies from Burkina Faso, Ghana and The Gambia.^4^, ^10^, ^11^

This article aims to (a) describe the overall prevalence of TTIs among donors attending one of the largest blood transfusion centres in Dar‐es‐Salaam, Tanzania, (b) compare the TTI prevalence between FRD and VNRD and (c) describe existing linkage to care for TTIs as defined by donor notification of a positive result and referral to respective clinical services.

## 2 METHODS

### 2.1 Study population

Between 1 October 2016 and 31 March 2017, a retrospective assessment of all blood donations at the blood transfusion centre at the Muhimbili National Hospital (MNH), Dar‐es‐Salaam was undertaken. As part of routine practice, all blood donors undergo systematic predonation counselling conducted by a trained nurse or social worker. This includes recording demographic information, previous donation history, TTIs risk factors and a basic medical assessment. In addition, candidates must be aged 18‐69 years, weight >50 kg and must not be anaemic (defined as ≥12.5 g/d for females and ≥13.5 g/d for males).

### 2.2 Serological methods used to detect HIV, HBV, HCV and syphilis

During the study period, all blood donors had samples tested locally for TTIs. The HIV antigen/antibody combination, HBV surface antigen (HBsAg) and anti‐HCV assays were performed on the Abbott ARCHITECT platform (Abbott Laboratories, Chicago, IL) in accordance with the manufacturer's specifications. Syphilis serology was performed using the syphilis 3.0 SD Bioline rapid diagnostic test (SD Biostandard Diagnostics Private Limited, Gurgaon, Haryana, India).

### 2.3 Data collection

For each donation entry, a range of data was recorded, including demographics (age and gender); donor status (first time or repeat); type of donation (voluntary or family/replacement); TTI status (HIV Ag/Ab, HBsAg, anti‐HCV Ab, syphilis serology); and evidence of the donors returning to collect the screening data from the unit.

### 2.4 Statistical analysis

Characteristics of the study participants were presented by mean and standard deviation (SD) for the continuous variables and percentage for the categorical variables. Continuous variables were compared using the student's *t* test and categorical variables using the chi‐squared test or Fisher's exact test where appropriate. Prevalence and their 95% binomial confidence intervals (CI) were calculated. Factors associated with TTI were identified using a logistic regression. We predetermined the following variables as potential determinants for TTI: age, sex, donor status (first time or repeat) and type of donation (voluntary or family/replacement). For all the variables, we systematically adjusted for potential distal determinants (age and sex), irrespective of the results of the univariable analyses. Factors with a *P*‐value of less than 0.05 were considered statistically significant. R statistical computing freeware version 3.4.3 was used for all analyses.

## 3 RESULTS

### 3.1 Blood donor demographics

Between 1 October 2016 and 31 March 2017, 6042 potential blood donors were registered in the MNH blood transfusion unit. Their characteristics are summarized in Table 1. Donors were predominantly males (n = 5383 (84.1%)), and the mean age was 34.7 (SD 9.8) years. FRDs accounted for the vast majority of donations (n = 5634 (88%)) of whom more than half (n = 3154 (56%)) were first‐time donors. Conversely, the VNRDs minority was overwhelmingly made up by repeat donors (n = 662 (86.7%)).

**Table 1:** Demographic characteristics of blood donors attending the transfusion unit

Variables	Total (n = 6402)	Family/Replacement donors (n = 5634)	Voluntary Nonremunerated donors (n = 763)	*P*‐value
Male sex, n (%)	5383 (84.1)	4722 (83.8)	656 (86.0)	0.1
Mean age, years (SD)	34.7 (9.8)	34.4 (9.6)	37.3 (10.5)	<0.001
First‐time donor, n (%)	3256 (50.9)	3154 (56.0)	101 (13.2)	<0.001

*P*‐values generated using chi‐squared test for categorical variables (sex and first‐time donor) and student's *t* test for continuous variable (age).

### 3.2 TTI prevalence

The overall TTI prevalence in was 8.4% (95% CI 7.8‐9.1), with HBV being the most prevalent infection (4.1% (95% CI 3.6‐4.6)), followed by syphilis (2.2% (95% CI 1.8‐2.6)), HIV (1.7% (95% CI 1.4‐2.0)) and HCV (1.0% (95% CI 0.7‐1.2)).

### 3.3 Factors associated with TTIs

Overall, TTIs were more than twice as likely in FRDs (9.0%, 95% CI: 8.3‐9.8) compared with VNRDs (4.1%, 95% CI: 2.8‐5.7, *P* < 0.001). The prevalence of TTIs was also higher in repeat donors (7.9%, 95% CI: 7.0‐8.9) than in first‐time donors (8.9%, 95% CI: 8.0‐10.0). The TTIs prevalence significantly differed according to the age groups, with the peak prevalence of 10.5% (95% CI: 9.1‐12.0) seen in the 35‐44 age group. There was no statistically significant difference in TTIs prevalence between men (8.7%, 95% CI: 7.9‐9.4) and women (7.3%, 95% CI: 5.7‐9.0) (*P* = 0.1).

The statistically significant difference in the prevalence of overall TTIs observed between FRDs and VNRDs remained for each category of infection, except for HCV, after adjusting for age and sex: HBV (4.4% (95% CI 3.9‐5.0) vs 1.8% (95% CI 1.0‐3.1), adjusted *P* = 0.002), HIV (1.8% (95% CI 1.5‐2.2) vs 0.5% (95% CI 0.1‐1.3), adjusted *P* = 0.008) and syphilis (2.3% (95% CI 1.9‐2.7) vs 1.4% (95% CI 0.7‐2.6), adjusted *P* = 0.03). HBV was also more common among first‐time donors compared to repeat donors (4.7% vs 3.4%, adjusted *P* = 0.003) and males compared to females (4.4% vs 2.8%, adjusted *P* = 0.02) (Table 2).

**Table 2:** Factors associated with, all TTIs, HBV, HCV, HIV and syphilis^a^

Variable	All TTIs			HBV			HCV			HIV			Syphilis		
	Prevalence (%) (95% CI)	Adjusted OR (95% CI)	*P*‐Value	Prevalence (%) (95% CI)	Adjusted OR (95% CI)	*P*‐Value	Prevalence (%) (95% CI)	Adjusted OR (95% CI)	*P*‐Value	Prevalence (%) (95% CI)	Adjusted OR (95% CI)	*P*‐Value	Prevalence (%) (95% CI)	Adjusted OR (95% CI)	*P*‐Value
Age (years)															
18‐24	5.3 (4.0‐6.8)	1.0	<0.001	2.5 (1.7‐3.7)	1.0	<0.001	1.0 (0.5‐1.8)	1.0	0.4	0.9 (0.4‐1.7)	1.0	<0.001	1.4 (0.8‐2.3)	1.0	<0.001
25‐34	7.5 (6.5‐8.6)	1.5 (1.1‐2.0)		4.9 (4.1‐5.8)	2.0 (1.3‐3.1)		0.8 (0.5‐1.2)	0.8 (0.4‐1.8)		1.2 (0.8‐1.7)	1.3 (0.7‐3.0)		0.8 (0.5‐1.3)	0.6 (0.3‐1.2)	
35‐44	10.5 (9.1‐12.0)	2.1 (1.6‐2.9)		4.9 (3.9‐5.9)	2.1 (1.3‐3.1)		1.3 (0.8‐1.9)	1.3 (0.6‐2.9)		2.5 (1.9‐3.3)	2.9 (1.5‐6.4)		2.4 (1.8‐3.2)	1.8 (1.0‐3.4)	
≥45	10.0 (8.3‐11.9)	2.0 (1.4‐2.8)		2.5 (1.7‐3.6)	1.0 (0.6‐1.7)		0.8 (0.4‐1.5)	0.8 (0.3‐2.0)		2.1 (1.3‐3.1)	2.4 (1.1‐5.5)		5.4 (4.2‐6.9)	4.2 (2.4‐7.8)	
Sex															
Female	7.3 (5.7‐9.0)	1.0	0.1	2.8 (1.8‐4.0)	1.0	0.02	0.2 (0.0‐0.7)	1.0	0.01	1.9 (1.1‐2.9)	1.0	0.6	2.6 (1.7‐3.7)	1.0	0.3
Male	8.7 (7.9‐9.4)	1.2 (0.9‐1.6)		4.4 (3.8‐4.9)	1.6 (1.1‐2.5)		1.1 (0.9‐1.4)	5.8 (1.8‐35.6)		1.6 (1.3‐2.0)	0.9 (0.5‐1.5)		2.1 (1.7‐2.5)	0.8 (0.5‐1.2)	
First‐time or repeat donor															
First time	8.9 (8.0‐10.0)	1.0	0.004	4.7 (4.0‐5.5)	1.0	0.003	1.0 (0.66‐1.4)	1.0	0.7	1.8 (1.4‐2.3)	1.0	0.2	2.0 (1.6‐2.6)	1.0	0.4
Repeat	7.9 (7.0‐8.9)	0.8 (0.6‐0.9)		3.4 (2.8‐4.1)	0.7 (0.5‐0.9)		1.0 (0.7‐1.4)	0.9 (0.5‐1.5)		1.6 (1.2‐2.1)	0.8 (0.5‐1.1)		2.3 (1.8‐2.9)	0.9 (0.6‐1.2)	
Family replacement or voluntary donor															
Family replacement	9.0 (8.3‐9.8)	1.0	<0.001	4.4 (3.9‐5.0)	1.0	0.002	1.1 (0.8‐1.4)	1.0	0.08	1.8 (1.5‐2.2)	1.0	0.008	2.3 (1.9‐2.7)	1.0	0.03
Voluntary	4.1 (2.8‐5.7)	0.4 (0.3‐0.6)		1.8 (1.0‐3.1)	0.4 (0.2‐0.7)		0.4 (0.1‐1.1	0.4 (0.1‐1.0)		0.5 (0.1‐1.3)	0.3 (0.1‐0.6)		1.4 (0.7‐2.6)	0.5 (0.3‐0.9)	

a OR adjusted for age and sex for all variables.

### 3.4 Donor notification

Overall, only 8.5% (95% CI 6.3‐11.2) of infected blood donors were notified of a positive result. Donors with HIV infection were most likely to be informed of a positive result (13.1% (95% CI 7.3‐21.0)), while patients with HBV were least likely to be informed (5.7% (95% CI 3.2‐9.3)) (Table 3).

**Table 3:** Proportion of donors with TTIs notified of a positive result

TTIs	Infected (n)	Notified (n)	Proportion (%) (95% CI)
Any TTI	540	46	8.5 (6.3‐11.2)
HBV	262	15	5.7 (3.2‐9.3)
HCV	62	7	11.2 (4.7‐21.9)
HIV	107	14	13.1 (7.3‐21.0)
Syphilis	139	15	10.8 (6.2‐17.2)

## 4 DISCUSSION

The increased requirements and shortage in supply of blood in Africa are well documented. The recent WHO global report highlights the disparity in availability of blood across the globe, with supplies of blood in Africa falling woefully shy of the recommended threshold of 10 units/1000 people.^3^ In Tanzania, the existing supply stands at 3.6 units/1000, with the majority of blood obtained through FRD.^7^ This is highlighted in our study, where close to 90% of all donations were from FRD. A recent review of blood donation practice in SSA reflected similar practice, with 24 out of 34 published articles reporting donor status either partially or completely relying on FRD or commercial donation.^12^ In contrast, the WHO global report on blood transfusion safety reported that 67% of blood donations from the African region were from VNRD.^3^

There has been a growing impetus to improve global blood transfusion safety, which includes improved screening for TTIs. As recently as 2004 a report commissioned on transfusion safety in Africa reported that 88.5% of blood donated across 40 different countries had not been reliably tested for HIV.^13^ In 2006, it was reported that only 40, 34 and 23 out of 46 African countries surveyed systematically screened all blood products for HIV, HBV and HCV respectively.^13^ Further improvement was seen by 2013 estimates, where less than 10% of blood was not screened for all TTIs.^3^ One of the most cited issues with universal TTI screening is guaranteed supply of testing kits.^4^, ^14^, ^15^ Innovations to simplify testing have overcome the potential shortage in kit provision, particularly in rural settings. Approaches assessing the performance of rapid diagnostic kits have shown that they are both feasible and acceptable in Ghana, Malawi and The Gambia.^4^, ^16^, ^17^, ^18^

A more controversial strategy intended to improve transfusion safety is a drive to shift the dependence of donation from FRD to VNRD through centralized blood transfusion services.^3^ There is a fine balance to be struck by restricting the supply of blood without compromising demand, particularly in SSA where in the majority settings the existing system is already stretched.^19^ The repercussions of adopting this policy have been documented in Malawi, where it is reported that per capita blood donations dropped between 2011 and 2014, resulting in two‐thirds of the national transfusion need being unmet.^20^ In addition, the economic impact of a VNRD system is a 4‐8‐fold increase in cost per unit of blood, which is of particular relevance to resource‐limited settings.^12^, ^21^

It has been previously argued that a direct comparison of TTI prevalence between FRD and VNRD without taking into account first‐time/repeat donor status introduces a significant selection bias. Allain and colleagues have previously described that TTI prevalence in first‐time VNRD is equivalent to FRD and thus have proposed that a more sustainable model for blood transfusion in SSA is one based on repeat donors, irrespective of VNRD or FRD status.^12^, ^22^ However, the findings from our study indicate that this notion may not be completely reflective in this cohort. We found that TTI prevalence was highest among FRD, irrespective of first‐time or repeat status (9.1% (95% CI 8.0‐10.3) and 9.0% (95% CI 8.0‐10.1) respectively). Although the proportion of first‐time VNRD was comparably small, the prevalence was lower among first‐time VNRD (6.9% (95% CI 2.8‐13.8), while repeat VNRD represent the lowest risk (3.6% (95% CI 2.3‐5.4) (Table 4).

**Table 4:** Comparison of TTI prevalence according to family/replacement donor or voluntary nonremunerated donor status and donation history

TTIs	First‐time family/Replacement donors	Repeat family/Replacement donors	First‐time voluntary Nonremunerated donors	Repeat voluntary Nonremunerated donors	*P‐value*
	Prevalence (%) (95% CI)	Prevalence (%) (95% CI)	Prevalence (%) (95% CI)	Prevalence (%) (95% CI)	
Any TTI	9.0 (8.0‐10.1)	9.1 (8.0‐10.3)	6.9 (2.8‐13.8)	3.6 (2.3‐5.4)	<0.001
HBV	4.8 (4.04‐5.56)	4.0 (3.2‐4.8)	4.0 (1.1‐9.8)	1.5 (0.7‐2.8)	0.001
HCV	1.0 (0.7‐1.4)	1.1 (0.8‐1.6)	0.0 (0.0‐3.6)	0.5 (0.1‐1.3)	0.4
HIV	1.8 (1.4‐2.4)	1.8 (1.3‐2.4)	0.0 (0.0‐3.6)	0.6 (0.2‐1.5)	0.06
Syphilis	2.0 (1.5‐2.6)	2.6 (2.0‐3.3)	3.0 (0.6‐8.4)	1.2 (0.5‐2.4)	0.09

*P*‐values generated using Fisher's exact test.

In particular, it is interesting to note that cases of HIV and HCV were more common in the FRD group compared with first‐time VNRD. In the case of HIV, this may represent a proportion of undisclosed infections given the existing stigma associated with HIV. While transmission of HCV through injecting drug use is an underappreciated problem,^23^ and since it is considered a taboo practice, it is also likely to be undisclosed. Conversely, HBV and syphilis appear to be relatively similar in both donor groups, which may underline a general lack of awareness of TTI status across all donor groups. Mode of transmission is also an important determinant. In Africa, HBV is transmitted early in life^24^ with a high risk of chronic infection as opposed to other TTIs. Therefore, it is expected that the prevalence in first‐time blood donors irrespective of VNRD or FRD status is similar. Interestingly, the HBV prevalence remains consistently high across all age groups. It is important to note that the HBV vaccine was only introduced in 2002, in Tanzania, thus it will be interesting to monitor HBV future prevalence, particularly among young first‐time donors, as this may provide some proxy for the impact of the vaccination program.

Although some guidance to contact tract donors with a positive result may exist, in practice, this is often challenging owing to a lack of resources, inadequate donor contact information and difficulties encountered by individuals who have to travel long distances to receive results in person. Thus, the existing donor notification system is firmly reliant on a donors desire to know their infection status. Despite the high prevalence of TTIs in our study, less than 10% of positive cases were notified of their infection. Interestingly, it appears that there is a comparable prevalence among first‐time and repeat FRD for all TTIs. Conversely, TTI rates, on the whole, are lower among first time as compared to repeat VNRD. However, in the case of HIV and HCV repeat VNRD have marginally higher rates of infection as compared to first‐time VNRD. Although it is likely that poor rate of notification is a prime factor to explain high levels of TTIs among repeat donors, additional factors including intentional failure to disclose diagnosis at counselling for fear of stigma and incident infections may also contribute. Systematic screening of donors provides an opportunity to diagnose and link newly infected cases to care. In the most recent global report on viral hepatitis, the WHO has specified that the referral into specialist services for positive blood donors with viral hepatitis must be practiced routinely.^5^ Similar policies exist for HIV, where the UNAIDS 90:90:90 objectives would benefit from donation centre referrals.^25^ Donor infection notification has been poorly described in SSA in existing literature; however, a report from Burkina Faso described that only 15% of blood donors infected with HBV or HCV were notified of a positive result.^10^ An improvement in notification would have a positive impact on waste of resources and reduce the proportion of donated units, which are discarded because of a positive result.

Our study has some limitations. Firstly, it reflects blood donation practice from a single urban centre in Tanzania. Thus, it may not reflect disease epidemiology and donation practices in other centres, particularly in more rural settings. Secondly, our deductions are limited by the design being retrospective, in particular, the identified TTI risk factors were limited to those covariates observed. Thirdly, first‐time VNRD constitute a small proportion of the total donor population, which reduces the confidence of the estimated TTI prevalence. As policy on donor recruitment remains a debated topic in SSA, further evaluation of prevalence using a larger sample of first‐time VNRD across Tanzania will be particularly valuable to elucidate the relevance of the findings from this study. Finally, in current practice, donors who are notified of a positive result may not be linked to care and specialist services. A report from Ghana suggested that linkage to care from transfusion services can be as low as 6% for HIV and 2% for HBV.^11^ Thus, further reports describing linkage pathways and treatment outcomes of patients successfully identified through this process are necessary.^10^ However, we can assume that linkage to care of positive donors in our setting is very poor given the low rate of notification.

Despite much higher prevalence of TTIs in FRD as compared to VNRD, our study highlights that the main blood transfusion centre in Tanzania is firmly reliant on FRD, which is in line with the existing blood transfusion practice in the majority of countries in SSA. There is an existing debate over the value of restricted donor selection in SSA, with fears that this would worsen the already threatened blood supply. A credible solution would be to have an inclusive donor policy, with an increased emphasis on systematic TTI screening, notification and linkage to care. This not only would maintain a safe supply of blood but would also simultaneously improve access to care for those with a positive TTI and eventually contribute to viral hepatitis elimination and control of other TTIs.

## CONFLICT OF INTEREST

None of the authors listed declare a conflict of interest related to this work.
